# *Astragalus* in the Prevention of Upper Respiratory Tract Infection in Children with Nephrotic Syndrome: Evidence-Based Clinical Practice

**DOI:** 10.1155/2013/352130

**Published:** 2013-04-09

**Authors:** Chuan Zou, Guobin Su, Yuchi Wu, Fuhua Lu, Wei Mao, Xusheng Liu

**Affiliations:** Nephrology Center, Guangdong Provincial Hospital of Chinese Medicine, No. 111 Dade Road, Guangzhou, Guangdong Province 510120, China

## Abstract

*Aims*. To explore whether *Astragalus* or its formulations could prevent upper respiratory infection in children with nephrotic syndrome and how best to use it. *Methods*. We transformed a common clinical question in practice to an answerable question according to the PICO principle. Databases, including the Cochrane Library (Issue 5, 2012), PUBMED (1966–2012.8), CBM (1978–2012.8), VIP (1989–2012.8), and CNKI (1979–2012.8), were searched to identify Cochrane systematic reviews and clinical trials. Then, the quality of and recommendations from the clinical evidence were evaluated using the GRADEpro software. *Results*. The search yielded 537 papers. Only two studies with high validity were included for synthesis calculations. The results showed that *Astragalus* granules could effectively reduce URTI in children with nephrotic syndrome compared with prednisone treatment alone (23.9% versus 42.9%; RR = 0.56 and 95% CI = 0.33–0.93). The dose of *Astragalus* granules was 2.25 gram (equivalent to 15 gram crude *Astragalus*) twice per day, at least for 3–6 months. The level of evidence quality was low, but we still recommended the evidence to the patient according to GRADEpro with the opinion of the expert. Followup showed the incidence of URTI in this child decreased significantly. *Conclusions*. *Astragalus* granules may reduce the incidence of URTI in children with nephrotic syndrome.

## 1. Background

Infection is one of the most common complications in children with nephrotic syndrome (NS). The annual incidence of invasive bacterial infection based on retrospective case series was shown to be about 1-2%, and the cumulative risk for the 10-year susceptibility period was around 10–20% [1].

In China, the incidence of nosocomial infection has been reported to be as high as 34–79% in children with nephrosis [2–4] and 22% in adults [5]. The most commonly reported infection accompanying nephrotic syndrome was upper respiratory infection (8.8–29.27%) [6–8]. Most of these infections were closely associated with frequent relapses in children with nephrosis [9], especially viral upper respiratory tract infections (URTI) [10, 11], which resulted in significant admission rates and healthcare costs.

There are several interpretations of this high risk of infection in NS, including urinary losses of factors B and D of the alternative complement pathway, impaired polymorph phagocytic function, edema, and secondary effects of corticosteroids and cytotoxic therapy [12, 13]. Efforts have been made to develop effective interventions dealing with this problem globally. To date, various prophylactic interventions have been used and/or recommended for reducing the risk of infection in nephrotic patients in clinical practice. These include chemoprophylaxis with antibiotics, pneumococcal vaccines, and immunoglobulin replacement therapies [1, 13]. However, the effectiveness of these prophylactic therapies has not been confirmed, and these treatments are quite expensive and still have some adverse effects [14].

The Chinese herbal medicine has been used to address infections for thousands of years. One representative medicine, *Astragalus* (*Huangqi*), has been the most studied. Many studies have reported that *Astragalus* or its formulations could prevent upper respiratory infection in immunocompromised patients [15, 16].

## 2. Clinical Question

An 11-year-old boy was referred to Professor Huang Chunlin's renal clinic because of repeated relapses of nephrotic syndrome. Nine years earlier, he had been diagnosed with primary nephrotic syndrome after the onset of limb edema. Prednisone at a dose of 60 mg/day was given and gradually withdrawn when complete remission was achieved after 1 month. However, he suffered his first relapse of nephrotic syndrome when he was on a prednisone dose of 15 mg/day due to an upper respiratory tract infection. Although the infection was controlled quickly, his nephrotic syndrome was not alleviated, forcing him to take the initial dose of prednisone (60 mg/day) again. Another complete remission was then achieved. In the subsequent 9 years, he experienced a total of eight relapses whenever the prednisone dose was reduced to lower than 20 mg/day. In particular in the last year, URTI occurred eight times, and NS relapsed three times, apparently triggered by URTI. Three months before this referral, he started taking prednisone at a dose of 60 mg/day for the same clinical situation described above, and it had been tapered gradually to 20 mg at the time of the visit. At that time, he presented with what appeared to be Cushing's syndrome: flushed face, moon face, buffalo hump, and complaining of feeling fatigued and sweaty. Physical examination showed congestion of the throat with swelling of the bilateral tonsils and purple striae all over the body, but no edema of the lower limbs. Laboratory investigations showed the following: 24 h urinary protein was in the normal range, serum immunoglobulin (IgG) slightly low, serum albumin 38 g/L, and serum creatinine 45 *μ*mol/L. Kidney ultrasonography showed no abnormality.

His mother had heard about *Astragalus* (*Huangqi*), a Chinese herb, which has effects in strengthening immunological function and preventing respiratory tract infection. She inquired about the effects and application of *Astragalus* for her son's situation.

The common clinical question was whether it would be effective to use *Astragalus* to prevent upper respiratory infection in children with nephrotic syndrome. What would be the expected effects and the preferred route of application (dose, course of treatment)?

The PICO question was formulated as follows: in patients or a population with primary nephrotic syndrome, were *Astragalus* formulations (as sole agents or in combination with other drug regimens) of value compared with a placebo or conventional treatment of NS in effectively preventing upper respiratory tract infections?

Thus,

P: children with primary nephrotic syndrome,
I: *Huangqi* or *Astragalus* formulations (either as sole agents or in combination with other drug regimens),
C: conventional treatment alone,
O: upper respiratory infection rate.

## 3. Search for Evidence

### 3.1. Inclusion Criteria

All randomized controlled trials (RCTs) evaluating the use of *Huangqi* or *Astragalus* as sole agents or in combination with other drug regimens compared with other drugs in preventing URTI in children (0–18 years of age) with primary NS were included. There was no restriction on population characteristics, language, or publication type. The primary outcomes were the incidence of URTI and adverse events. Duplicate publications reporting the same groups of participants were excluded.

### 3.2. Search Methods for Identification of Studies

We designed a search filter restricted to articles relevant to the PICO question within PUBMED from (1966–2012.8), Embase.com (1980–2012.8), Cochrane Library (Issue 5, 2012), The Chinese Biomedicine Database (CBM from 1978–2012.8), WeiPu Chinese Scientific Magazine Database (VIP; 1989–2012.8), and China National Knowledge Infrastructure (CNKI; 1979–2012.8).

The search used a combination of search terms using relevant synonyms for the domain: “kidney diseases,” “glomerulonephritis,” “nephrosis,” “minimal change nephropathy,” “nephropathy,” “*Huangqi*”, “*Astragalus* membranaceus,” “*Astragalus*,” “respiratory tract infection,” and “upper respiratory infection.” The search was limited to clinical trials and Cochrane systematic reviews. We first tried to construct search strategies using PICO. However, the results were limited, and not all were relevant. Thus, we modified our search strategies using only PI to cover most relevant studies.

The titles and abstracts of all published articles obtained from these search strategies were examined to determine whether they were applicable to the purpose of this study.

### 3.3. Data Collection

Titles and abstracts from the preliminary search results were checked independently by two authors (Chuan Zou and GuoBin Su) to identify clearly irrelevant studies that needed to be discarded. Full texts of the remaining studies were evaluated to judge whether the inclusion criteria were met. If information on key points was unclear, the relevant original authors of the articles were contacted by telephone for clarification.

### 3.4. Data Extraction

Data extraction was conducted on full-text copies of all included trials using a data extraction form designed for the purpose. Data regarding the characteristics of the study, including the sample size, age, disease period, and clinical features of the children with NS, were gathered. The types and doses of different *Huangqi* formulations, followup time, side effects, and primary outcomes were independently abstracted from the remaining articles by two authors (Chuan Zou and GuoBin Su). Disagreements were resolved by discussion and consensus, mediated by a third author (XuSheng Liu).

### 3.5. Quality Assessment

The risk of bias in individual studies was assessed by the GRADE approach across five aspects: sequence generation, allocation concealment, blinding, incomplete outcome data, selective outcome reporting, and other potential biases. The GRADE approach was used to evaluate the quality of evidence, and the results are presented in the Summary of Findings table. The quality rating for studies assessed using the GRADE approach has four levels: high, moderate, low, or very low.

### 3.6. Data Analysis

Relative risk was calculated with the GRADEProfiler 3.2.2 (GRADE Working Group 2004–2007) and is shown in the GRADE Quality Assessments tables. A process was designed to combine data across studies to perform a meta-analysis when *Huangqi* or *Astragalus* was studied as the sole constituent or in combination with other regimens, regardless of the form of the agents (liquid, powder, granule, or pill). If the main effects of the whole compound in the studies were similar, data were combined. The fixed-effect mode was used to combine dichotomous data if homogeneity was found. If heterogeneity was found, the random-effect mode was used.

## 4. Results of Search for Evidence

### 4.1. Search Result

**Figure 1:**
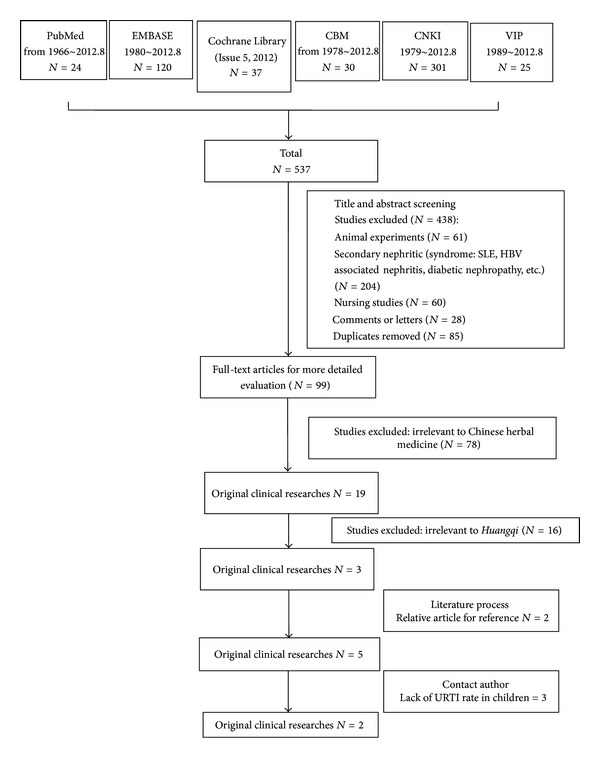
Search process: flow diagram of included and excluded studies. A search for relevant studies was performed using the PubMed database, Embase, Cochrane library, CNKI, CBM, and VIP subsequently filtered out based on the inclusion/exclusion described.

Our search yielded 24 articles in PubMed, 120 in Embase.com, 37 in Cochrane Library, 30 in CBM, 301 in CNKI, and 25 in VIP (for search strategies, see Table 1). Upon screening, five original clinical research articles [17–21] and two Cochrane systematic reviews [14, 16] remained for further analysis (for search and screening process, see flow chart, Figure 1).

Yuan et al. 2004 [21] compared* Huangqi* and *Hongzao* (red Chinese date) with placebo (*n* = 60) in children and adults (aged 8–60 years old). The study only included patients with refractory nephrotic syndrome (mesangial proliferative glomerulonephritis). The paper did not describe the baseline decline in renal function in the patients. The control group received prednisone treatment alone. The experimental group received *Huangqi* and *Hongzao *in addition to the drugs the control group received. The study showed that, compared with placebo, the *Astragalus* formulation (with *Hongzao*) significantly reduced the occurrence of respiratory tract infection (RR 0.27, 95% CI = 0.08–0.88) [16].

*Astragalus* granules significantly reduced the number of nosocomial infections or unspecified infections in children with nephrotic syndrome (two studies [17, 18], 130 participants: RR 0.62, 95% CI = 0.47–0.83; *P* = 0.001, *I* ^2^ = 0%) [14] and in adults (J. Zhang and Y. Zhang 2008 [20], RR 0.55). However, these studies did not report the specific respiratory tract infection rate.

Xie and Peng 2010 [19] compared standard prednisone treatment + *Huangqi* extract (by boiling *Huangqi* (*Astragalus*) 700 g with water 5600 g three times, 90 min each, and concentrating the solution to 700 mL, 10 mL t.i.d. p.o. for patients) with standard prednisone treatment alone (*n* = 64) in children and adults (aged 13.5–45 years old) and followed them for 1 year. The study only reported infection times in 1 year (intervention group: 2.57 ± 0.63 times/year versus control group: 5.32 ± 1.31 times/year) and did not describe the URTI rate.

We also identified two Cochrane reviews related to *Astragalus* and intervention for preventing infection in nephrotic syndrome. Yuan et al.'s review [16] covered three studies (*n* = 28): one [21] reported that *Huangqi* and red Chinese date reduced some adverse reactions (Cushing's syndrome: RR 0.55, 95% CI = 0.32–0.94; hormone withdrawal syndrome (HWS): RR 0.58, 95% CI = 0.39–0.85; respiratory tract infection: RR 0.27, 95% CI = 0.08–0.88). Wu et al.'s review [14] included 12 studies conducted in China that included 762 children with nephrotic syndrome. With four positive interventions, that is, intravenous immune globulin (IVIG), Bacillus Calmette-Guerin (BCG), *Astragalus*, and TIAOJINING, two studies [17, 18] reported that *Astragalus* granules showed a significantly beneficial effect (RR 0.62, 95% CI = 0.47–0.83) in reducing the risk of infection in children with nephrotic syndrome. However, neither study specifically reported the respiratory infection rate.

We did not include J. Zhang and Y. Zhang (2008) [20] or Xie and Peng (2010) [19] because the populations they studied contained not only children. We also did not include Yuan et al. (2004) [21], even though it was the only study that reported the URTI rate, because the study included both children and adults. When we tried to contact the authors to get the number of children (aged below 18) with URTI, we received no response.

We managed to get the URTI rate in the two remaining studies [17, 18] by contacting the authors by telephone or email. Thus, only J. Chen and S. Q. Chen (2008) [18] and Kang (2005) [17] were included in our further evaluation and analysis. The characteristics of the included studies are summarized in Tables 2 and 3.

### 4.2. Quality of Evidence

The quality of methods and reporting of results in the remaining five articles were appraised critically for quality of methodology, design, and data analysis using GRADE. The quality of these studies was low (Table 5). All the studies included were unclear in terms of risk of bias; the papers did not provide bias information regarding the study design. Thus, we assumed that the potential limitations were likely to lower the confidence in estimation of effects. No study reported information about the randomization sequence generation or blinding (Table 4). The RR for *Huangqi* intervention versus conventional treatment alone was 0.56 (95% CI = 0.33–0.93; *P* = 0.10; Table 5).

## 5. Application of Evidence and Followup

According to the two studies included [17, 18] and the experience of Professor Huang Chunlin, we administered *Astragalus* granules (the *Astragalus* was from Shanxi Province, China). The origin of the herb was authenticated, and the material was examined for microorganisms, heavy metals, and pesticides, according to accepted standards (good manufacturing practices, GMP) in China. The *Astragalus* was found to be of good quality, and the crude *Astragalus* had been extracted by TianJiang Pharmaceutical Co. Ltd., China. The *Astragalus* was extracted at a ratio of 1.5 g from 10 g of crude *Astragalus*. Freeze-dried extracts of *Astragalus* were analyzed by high-performance liquid chromatography using Astragaloside IV as an active component. 

The patient was administered 2.25 g *Astragalus* granules (equivalent to 15 g crude *Astragalus*) twice/day for 2 years. During the *Astragalus* granule treatment period, prednisone was tapered gradually (prednisone taper, 2.5 mg/month). When the patient had URTI, he was asked to stop taking *Huangqi* granules and to maintain his prednisone dosage. After 3 months of *Astragalus* granule treatment, he felt less fatigued and sweaty, and his serum immunoglobulin G reached normal levels. During the maintenance period on prednisone (10 mg/d), he suffered from URTI once, and this was alleviated in 3 days without relapse of NS. He had URTI twice; his symptoms were mild and lasted for no more than 7 days, without relapse of NS, 1 year after the cessation of prednisone. To date, he has had a total of three episodes of URTI in 2 years, compared with 8 times per year before *Astragalus* treatment, and no relapse of NS, compared with three relapses in 1 year before *Astragalus* treatment.

## 6. Discussion

### 6.1. Mechanism of *Astragalus* in Preventing URTI

Before 1940, mortality in children with nephrotic syndrome reached 40%, due, mainly, to infections [22]. Although mortality declined dramatically after the introduction of glucocorticoids and antibiotics, infections remained the major cause of death in children with nephrotic syndrome [23, 24]. The severe infections leading to death included sepsis, peritonitis, and pneumonia [25]. Acute URTI may not threaten the lives of nephrotic syndrome patients; however, it may be associated with relapse and acute exacerbation of nephrotic syndrome [5]. Its mechanism(s) involve the following aspects: (1) a decrease in immunoglobulins [26], (2) dysfunction in specific antibody production [27], (3) decreases in complement bypass factors B and D [28, 29], and (4) immunosuppressive therapies.

*Astragalus* has been shown to be able to correct the problems causing susceptibility to URTI in nephrotic patients. For example, *Astragalus* decreased soluble IL-2 receptor (SIL-2R) and IL-8 levels, while increasing IgA, IgM, and IgG levels in patients suffering from recurrent URTI [30]. Experimental studies found that oral administration of an *Astragalus* formulation (Bu-Zhong-Yi-Qi-Tang, 100 mg/kg per day) to young BALB/c mice enhanced influenza virus-specific IgA antibody titers significantly in the nasal cavity. This may be one of the mechanisms by which *Astragalus* and its formulations reduce influenza virus infection [31].

*Astragalus* was also found to reduce overinhibition by immunosuppressants. In vitro studies showed that *Astragalus* reversed the inhibition of cyclophosphamide on T lymphocytes in experimental rats [32, 33]. In China, *Astragalus* and its formulations have been used widely in patients on immunosuppressive treatment. *Astragalus* inhibits common viruses, such as coxsackievirus [34] and influenza virus, and common bacteria, such as nonhemolytic streptococcus, pneumococcus, and *Staphylococcus aureus* [35], that can cause upper respiratory tract infections.

Based on these findings, *Astragalus* would seem to have a good biological basis for use in preventing URTI in nephrotic patients.

### 6.2. Evidence-Based Clinical Practice

According to the GRADE method of bias evaluation, there were potentially large biases in the included studies, which could have had an impact on the outcome. For example, there was no description of adequate sequence generation, allocation concealment, or blinding, which could have produced bias in selection, implementation, and measurement. All eligible studies were carried out in China and published in Chinese; thus, positive results were more likely to have been reported than negative results [36]. For these reasons, the risk of bias in these trials was potentially serious, and the level of evidence by GRADEpro was low.

Although the level of evidence for using *Astragalus* with the patient was low, we had the aid of the GRADE instruments to make the clinical decision. First, the GRADE tools consider the recommendation strength, reflecting how well we could determine whether the intervention would do more good than harm [37]. The benefits of intervention include lowering morbidity and mortality, improving the quality of life, and reducing medical burden, whereas the disadvantages are the opposite outcomes. Our study found that Radix Astragali plus prednisone could significantly reduce the incidence of URTI in children with nephrotic syndrome compared with prednisone treatment alone, while having no side effects in most cases.

Second, because each treatment has its own advantages and disadvantages, it is important for doctors to make clinical decisions by evaluating benefit, risk, and convenience from the patient's point of view [38]. The patient and his relatives obtained relevant information on the Internet about the ability of Radix Astragali to reduce respiratory infection, which was confirmed by its use in other patients, so they were eager for the patient to have Radix Astragali treatment, and they wanted to know about the direction and course of treatment.

Finally, another key factor in clinical decision making is the cost. The possibility of a strong recommendation for a very good treatment can be reduced because of its high cost and unavailability. Thus, one of the crucial factors in recommendation strength is evidence regarding the conditions and demands of its practical use [39]. *Astragalus* therapy is much cheaper than other methods commonly used in China to prevent URTI in nephrotic syndrome, such as injections of gamma globulin or thymosins. Thus, it can be used with the vast majority of patients with nephrotic syndrome.

To summarize, we decided to administer Radix Astragali to the patient to prevent URTI, and the dose and course were referred to an expert for an opinion. According to Professor Huang's experience of syndrome differentiation in Chinese medicine (CM), the outstanding symptoms of the boy were fatigue, hidrosis, and being prone to suffer from upper respiratory tract infection, indicating a lung Qi deficiency. *Astragalus* membranaceus was the right herbal medicine for this syndrome. From the perspective of modern medicine, the serum IgG of the patient was slightly low, showing a decline in humoral immune function, and this may have been related to the frequent occurrence of upper respiratory tract infections. *Astragalus* can improve immune function and enhance the serum level of IgG. Thus, the use of *Astragalus* in the patient could prevent the relapse of NS through reducing the incidence of upper respiratory tract infection. The dose and duration of *Astragalus* treatment for the patient were determined from reports of clinical trials and the experience of Professor Huang. The *Astragalus* granules were from a pharmaceutical company that was GMP compliant. They were administered to the patient, while prednisone was slowly tapered, though maintained for a long enough time. When an upper respiratory tract infection occurred, manifested as a sore throat and fever, the *Astragalus* granules were stopped temporarily and then restarted when the infection was controlled and the symptoms disappeared. After 3 months of therapy, the syndrome of lung Qi deficiency in the patient was obviously improved, and the serum IgG returned to normal levels. In the 2-year period of prednisone maintenance and discontinuance after administration of *Astragalus*, his incidence of upper respiratory tract infection was diminished significantly (1.5 times per year versus eight times per year), and there was no relapse of NS.

## Figures and Tables

**Table 1:** Search strategies.

Database	Searching strategies	Hits
PubMed (1966~2012.8)	P: (“Kidney diseases” [Mesh] OR “Glomerulonephritis, Membranous” [Mesh] OR “Glomerulonephritis, IGA” [Mesh] OR “Glomerulonephritis” [Mesh] OR “Glomerulonephritis, Membranoproliferative” [Mesh] OR “Glomerulosclerosis, focal segmental” [Mesh] OR “Glomerulonephritis” [text word] OR “nephrosis, lipoid” [MeSH terms] OR (“nephrosis” [all fields] AND “lipoid” [all fields]) OR “lipoid nephrosis” [all fields] OR (“minimal” [all fields] AND “change” [all fields] AND “nephropathy” [all fields]) OR “minimal change nephropathy” [all fields] OR “nephropathy” [text word] OR “Glomerulo*” [text word] OR “nephrotic syndrome” [Mesh] OR “nephr*” [text word]) I: “Huang Qi” [supplementary concept] OR “Huang Qi” [all fields] OR “huang qi” [all fields] OR “astragalus membranaceus” [MeSH terms] OR (“astragalus” [all fields] AND “membranaceus” [all fields]) OR “astragalus membranaceus” [all fields] C: other therapies O: (“respiratory tract infections” [MeSH terms] OR “respiratory tract infections” [all fields] OR “upper respiratory infection” [all fields])	P + I = 59 P + I Filters: Humans = 24 P + I + O = 0

EMBASE (1974~2012.8)	P: #1 OR #2 OR #3 OR #4 OR #5 OR #6 #1 nephrotic AND (“syndrome”/exp OR syndrome) #2 “kidney”/exp OR kidney AND (“diseases”/exp OR diseases) #3 “glomerulonephritis”/exp OR glomerulonephritis #4 “glomerulosclerosis”/exp OR glomerulosclerosis #5 “nephrosis”/exp OR nephrosis #6 “nephropathy”/exp OR nephropathy #5 “nephrosis”/exp OR nephrosis #4 “glomerulosclerosis”/exp OR glomerulosclerosis I: #1 OR #2 OR #3 OR #4 OR #5 #1 “astragalus” #2 “huangqi” #3 “Astragalus extract”/exp #4 “Astragalus membranaceus”/exp #5 “Astragalus membranaceus extract”/exp C: other therapies O: #1 respiratory AND (“infection”/exp OR infection) #2 “respiratory tract infections”/exp OR “respiratory tract infections”	P + I = 266 Limited human 120 P + I + O = 17

Cochrane Library (Issue 5, 2012)	Searching field: title, abstract, or keywords P: “Kidney diseases” OR “Membranous Glomerulonephritis” OR “Glomerulonephritis” OR “lipoid nephrosis” OR “minimal change nephropathy” OR “minimal change disease” OR “nephrotic syndrome” OR “nephr*” I: “Huang Qi” OR “astragalus membranaceus” C: other therapies O: (“respiratory tract infections” OR “respiratory tract infections” OR “upper respiratory infection”)	P: 37 P + I = 3

CBM (1978~2012.8)	Subject or title or abstract: Nephritic syndrome AND respiratory infection	30

CNKI (1979~2012.8)	Subject or title or abstract: Nephritic syndrome AND respiratory infection	301

VIP (1989~2012.8)	Title or keyword: Nephritic syndrome AND respiratory infection	25

*Refers to searching for all terms that begin with a word, enter the word followed by an asterisk (*).

**Table 2:** Characteristics of the included studies.

	Study design	Population	Intervention	Comparison	Duration	Outcome	Followup	Adverse reaction
Kang, 2005 [17]	Parallel RCT *N* = 38	Age I: 1.5 to 6 y C: 1.8 to 7 y	STD prednisone + Huangqi (Astragalus) granules Dose: 15 g b.i.d.p.o.			Infection (I: 14/22, C: 15/16)		
				STD prednisone	3 to 6 months	URTI (I: 8/22, C: 9/16)	Not mentioned	NS
						Relapse (I: 10/22, C: 13/16)		

J. Chen and S. Q. Chen, 2008 [18]	Parallel RCT *N* = 92	Age I: 2.0 to 13.2 y C: 2.1 to 13.7 y	STD prednisone + Huangqi (Astragalus) granules (oral) Dose: 7.5 g bid (<3 years); 10 g bid (3 to 6 years); 15 g bid (>6 years)			Infection (I: 14/45, C: 28/47)		
				STD prednisone	3 months	URTI (I: 8/45, C: 18/47)	8 months	NS
						Relapse (I: 9/45, C: 23/47)		

STD prednisone: initial dose 1 mg/kg/d for 8–12 weeks. Every other day for 6 months. When urine protein is negative, decreased 5 mg/wk until reduced dose tapered gradually to 0.4 mg/kg/d over 6 months and maintained at this dose for 12–18 months. Finally, tapered gradually until withdrawal to 0.5 mg/kg/d, then 1 mg/kg every other day for 6 months.

STD prednisone + CTX: initial prednisone dose 1 mg/kg/d plus CTX 50 mg b.i.d.p.o. (total in 8 mg).

URTI: upper respiratory tubular infection; I: intervention; C: comparision.

**Table 3:** Characteristics of the included Cochrane reviews.

	Intervention	Comparision	Number of RCTs	Participants
Wu et al., 2012 [14]	IVIG, thymosin, OTF, MPT, BCG, Lantigen B, TIAOJINING, Huangqi granules + baseline treatment	Baseline treatment	12	762

Yuan et al., 2008 [16]	Huangqi injection, Huangqi and red Chinese, Huangqi and Danggui + baseline treatment date	Baseline treatment	3	128

IVIG: intravenous immunoglobulin; OTF: oral transfer factor; MPT: mannan peptide tablet; BCG: Bacillus Calmette-Guerin (BCG) vaccine injection; and baseline treatment: STD prednisone.

**Table 4:** Quality assessment of the included studies.

	Concealment of allocation	Randomization sequence generation	Blinding	Selective reporting	Intention-to-treat analysis	Level of evidence
Kang, 2005 [17]	NS	Unclear^1^	No	Not mentioned	Not mention	Unclear risk

J. Chen and S. Q. Chen, 2008 [18]	NS	Quasi-randomization^2^	No information	Not mentioned	Not mention	Unclear risk

^1^The study did not provide the information on the method of random sequence generation and only presented the data that participants with nephrotic syndrome were randomly allocated to treatment and control groups.

^2^The patients with nephrotic syndrome were allocated to the treatment and control groups according to the order of admission.

**Table 5:** Grade assessment of studies. Question: should Huangqi versus conventional treatment be used for upper respiratory tract infection? Settings: parallel RCT.

Upper respiratory tract infection rate												
Quality assessment							No. of patients		Effect		Quality	Importance
No. of studies	Design	Risk of bias	Inconsistency	Indirectness	Imprecision	Other considerations	Huangqi	Conventional treatment	Relative (95% CI)	Absolute
							16/67 (23.9%)	27/63 (42.9%)		19 fewer per 100 (from 3 fewer to 29 fewer)		
2	Randomised trials	Serious^1^	No serious inconsistency	No serious indirectness	Serious^2^	None		10%	RR 0.56 (0.33 to 0.93)	4 fewer per 100 (from 1 fewer to 7 fewer)	⨁⨁○○ Low	Critical
								40%		18 fewer per 100 (from 3 fewer to 27 fewer)		

^1^Studies were at unclear risk of bias, for the paper did not provide the bias information of the study design. So we determine the potential limitations are likely to lower confidence in the estimate of effect.

^2^Total number of events is less than 300, and a relative risk reduction (RRR) or relative risk increase (RRI) is greater than 25%.
